# Enhancing Keratoconus care in a public healthcare system in KwaZulu-Natal, South Africa

**DOI:** 10.4102/phcfm.v17i1.4840

**Published:** 2025-10-21

**Authors:** Nonkululeko M. Gcabashe, Vanessa R. Moodley, Sanele Buthelezi, Rekha Hansraj

**Affiliations:** 1 Department of Optometry, School of Health Sciences, University of KwaZulu-Natal, Durban, South Africa; 2 Department of Optometry, Faculty of Health Sciences, University of Johannesburg, Johannesburg, South Africa

**Keywords:** health system, enabling healthcare, healthcare strengthening, keratoconus, public sector

## Abstract

**Background:**

Keratoconus (KC) is a self-limiting corneal ectasia with an unknown definitive aetiology. It presents as a corneal thinning disorder that results in myopia and irregular astigmatism and, if left untreated, leads to visual impairment.

**Aim:**

This study aimed to understand the current enabling factors and challenges experienced in diagnosing and managing KC.

**Setting:**

The study setting involved public health facilities in KwaZulu-Natal (KZN).

**Methods:**

A qualitative design within an interpretive paradigm was conducted on 22 participants who were purposively selected. Interviews were conducted and data on the participant demographics and clinical and operational systems related to KC diagnoses and management were extracted.

**Results:**

Among participants, 55% were from the eye care field, while 23%, 14% and 9.1% were from the supply chain management, finance and operations departments, respectively. A lack of clinical equipment, inadequate clinical infrastructure, a shortage of eye care personnel and insufficient funding emerged as barriers to the provision of comprehensive eye care to patients diagnosed with KC.

**Conclusion:**

The study highlighted that the minimum standard of care expected for patients diagnosed with KC is not being met. It thus recommended that a new policy be developed or existing policies for the diagnosis ad management be reviewed.

**Contribution:**

The findings of this study are expected to inform and enhance the provision of eye care services for patients diagnosed with keratoconus within the public healthcare system.

## Introduction

The health system has been defined by the World Health Organization (WHO) as ‘all organisations, institutions and resources devoted to producing action whose primary intent is to improve health’.^1^ South Africa (SA) has an overarching national health department with provincial governance departments established in each province. Healthcare is delivered via two parallel systems, namely, private and public.^2^ An effective healthcare system is essential and healthcare should be provided to every person to ensure a good quality of life, as it is a significant resource for personal, economic and social development.^3^ The health system can be structured at different levels of delivery, such as the primary, district and national healthcare levels, as guided by the WHO health system framework. The WHO framework comprises six core components, namely, service delivery, information and research, medical products, technologies, health workforce, healthcare financing, and leadership or governance.^4^ The South African healthcare system is based on a hierarchical referral from primary health care clinics to district, regional, tertiary and quaternary hospitals. The health system in the province of KwaZulu-Natal (KZN) in SA is structured within the national health guidelines using the district-based approach.^2^

Eye care services are facilitated or delivered by different cadres of clinical and management teams, such as ophthalmic nurses, medical officers, supply chain managers, finance personnel, ophthalmologists and optometrists, the latter being the leading role players. The WHO recommends a minimum practitioner-to-patient ratio of 1:100 000 for optometrists or refractionists and 1:250 000 for ophthalmologists.^5^ However, in developing countries, these recommended ratios may not be met because of the unequal distribution of optometrists between urban and rural areas.^6^ Although more than twice the number of ophthalmologists, the 63 optometrists currently employed within the public sector still need to be considered as being low to meet eye care needs (Mthethwa S, personal communication, June 2023). It is noteworthy that practitioner-to-patient ratios cannot be viewed in isolation as the right to health is meaningless without providing good quality care, which is foundational to improving health systems.^7^

The Department of Health (DoH) in KZN is responsible for provincial and district healthcare services and, therefore, is the overarching authority for the health workforce within the public sector, including managing the provincial health budget. A healthcare delivery system cannot function without a well-designed and operated administration, which includes supply chain, asset and finance management. The acquisition of medicines or medical products and the procurement and maintenance of equipment is also included in health administration. The head of supply chain management (SCM) keeps track of logistics, analyses employees’ operational performance and updates the assets and inventory of the facility. Moreover, the aforementioned supervises employees to ensure effective collaboration between the hospital and the suppliers to meet the quality and safety standards of the health department.^8^ The finance team, both at the national and provincial hospital levels, is responsible for supporting the healthcare institution’s overall financial well-being to the ultimate benefit of the patients that it serves. They must also be familiar with the *Public Finance Management Act (PFMA)*, SCM framework and reports for the different committees (cash flow and finance). In addition, they coordinate training and guidance to staff members (including eye care personnel), among other responsibilities.

Optometrists in the public sector are a part of the human resource personnel for eye care delivery. They are employed by the DoH under three grades based on their respective levels of experience.^8^ They must demonstrate a sound understanding of optometry techniques and practices and participate in outreach activities, including functioning within multi-disciplinary teams and linking with outside service providers, non-governmental organisations (NGOs) and organisations for persons with disabilities to provide comprehensive, holistic care. Furthermore, they foster eye care delivery based on the Batho Pele Principles and Human Rights Ethics.^7^ The input of optometrists into Human Resource (HR) planning, as well as securing and maintaining all necessary equipment, is vital in directives aimed at reducing the burden of visual impairment (VI).^8^

Optometrists and ophthalmic nurses serve as primary eye care providers in many remote areas where other eye care cadres are not easily accessible.^9,10^ Therefore, optometrists play a vital role in detecting and managing ocular diseases, including keratoconus. Early detection and management of the ocular disease will assist in reducing the burden of VI, which is a public health concern, considering that people living with VI tend to have a reduced quality of life.^11^ However, SA has been reported to have multiple challenges concerning the organisation of care and delivery of eye care services, which include vision assessments and the provision of optical corrections.^12,13^

Effective management of any ocular disease needs early detection and accurate diagnosis, which requires knowledgeable practitioners with access to relevant equipment and appropriate treatment options that may include prescribing optical devices (there are different strategies for effective treatment). Also required is an emphasis on the importance of regular eye examinations and follow-up so that symptoms of eye disease do not go unnoticed. Eye care education should be undertaken to prevent vision loss in those diagnosed with eye disease. For effective keratoconus (KC) management, a suitably trained eye care professional must be supported by an administration team, including human resources, finance and supply chain management teams. The optometrist enables the detection and management of KC; however, this will not be possible if the required resources are unavailable. Challenges to resources needed to detect, diagnose and manage KC vary from lack of clinical equipment to shortage of funds and/or personnel to deliver that service. Optometrists have long been responsible for the non-surgical visual rehabilitation of keratoconic patients and are trained in all aspects to manage these patients. Management of KC requires well-trained clinicians with access to the relevant equipment and adequate available funding to manage the disease with spectacles, contact lenses and surgical options.

With the sudden increase in the number of patients diagnosed with advanced KC presenting to public health sector facilities, there is a need to develop a framework or guideline for early detection and management. Anecdotal evidence suggests that the patients at these public health sites, particularly those in the early stages of KC, when interventions may produce better outcomes, might still need to be diagnosed. Therefore, this study set out to understand the current enabling factors and possible challenges in diagnosing and managing KC in public health facilities in KZN.

## Research methods and design

This study followed a qualitative design within an interpretive paradigm.^14^ Purposive sampling was used to recruit a total of 22 participants who responded positively to the invitation to participate. The participants included senior optometrists and/or clinic managers, operations managers, procurement officers, supply chain management managers and finance managers, all in the different facilities with functioning eye care departments but within the public sector in the province of KZN. The facilities that were considered for inclusion in the study were McCord Provincial Eye Hospital, Prince Mshiyeni Memorial Hospital, RK Khan Hospital, GJ Crookes Hospital, Port Shepstone Regional Hospital, Ngwelezana Hospital, Madadeni Hospital Harry Gwala Regional Hospital (formerly Edendale Hospital), General Justice Gizenga Mpanza Regional Hospital (GJGM) (formerly Stanger Hospital) and Mosvold Provincial Hospital, as well as the provincial office in KZN.

The invitation to participate in the study was sent via email and social media platforms. Semi-structured interviews, guided by an interval schedule, were used to explore three areas. Section one ascertained demographic information such as age, race, district, role or position in eye care, highest qualification and work experience. Sections two and three focused on eye care human capacity, eye care infrastructure, eye care operational systems and available protocols or guidelines. Probe questions in each area were used to gather the demographic data and explore their opinions, knowledge and experiences about the provision of eye care within the public sector in the different facilities, focusing on diagnosing and managing patients with KC at the facilities. Data analysis was approached thematically, beginning with coding, pattern recognition and identification of themes. Informed consent and relevant gatekeeper permissions were acquired before the commencement of the study. Individual confidentiality was ensured by personal data being de-identified through study numbers being applied to each participant.

### Ethical considerations

Ethical approval was granted by the Biomedical Research Ethics Committee at the University of KwaZulu-Natal (BE332/19) and the KZN Department of Health. Informed consent was sought from participants before the interviews.

## Results

### Demographic profile of participants

Twenty-two participants participated in the study, the majority of whom were female (72.7%), were black people (72.7%) and aged between 31 years and 40 years (40.9%). There were more participants from the optometry department (59.1%) compared to finance (13.6%), supply chain asset management (9.1%) and maintenance (18.2%) departments (Table 1). Most participants had been in their current positions for 6 months to 34 years. The districts represented by the participants in this study were eThekwini, uGu, iLembe, King Cetshwayo, aMajuba, uMkhanyakude and uMgungundlovu.

**TABLE 1:** Current positions held by the participants at the public sector health facilities.

Occupation titles	Number of participants	%
Head Optometrist	7	31.8
Resident Optometrist	5	22.7
Finance Manager	1	4.5
Finance Director	1	4.5
Supply Chain Asset Manager	1	4.5
Supply Chain Management Clerk	2	9.1
Supply Chain Management Officer	1	4.5
Operations Manager	1	4.5
Supply Chain Management Manager	1	4.5
Assistant Director of Finance	1	4.5
Deputy Director of Chronic Disease, Geriatric and Eyecare in KZN	1	4.5

KZN, KwaZulu-Natal.

**FIGURE 1:**
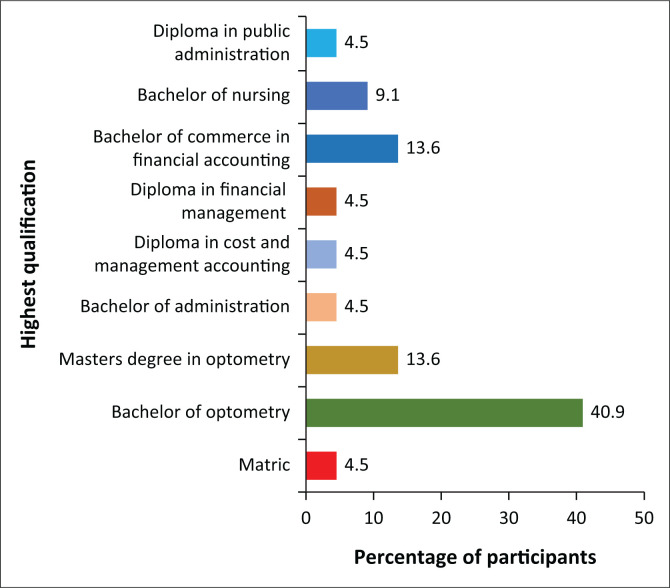
Highest qualification held by the participants.

The highest qualification held by most participants was a degree certification followed by a diploma, as shown in Figure 1. The participants also indicated having more than 5 years of experience in their current or similar field. The participants from the supply chain management had the highest number of years of experience (average of 19.4 years) compared to the eye care professionals (average of 16.7 years).

### Eye clinic systems

The qualitative results presented in the next section are from the key informants who participated in the study. Following the thematic analysis of the transcripts, three major themes were identified, namely, human capacity, infrastructural capacity and operational capacity.

#### Theme 1: Human capacity in keratoconus management

The focus of Theme 1 is on the human capital-related issues in the eye clinic facilities as experienced by the participants regarding the diagnosis and management of KC. Two subthemes described factors influencing this aspect of the eye clinic systems and the management of KC, for example, human resource distribution and continuous professional development.

**Human resource distribution:** The eye care professionals practising within the KZN DoH eye clinics include optometrists, ophthalmic nurses, medical officers and ophthalmologists. However, the uneven distribution of the cadres in each eye clinic and the inadequate number of eye care professionals, as noted in the following statement were apparent:

‘We do not have enough health professionals because all doctors, nurses, and clinicians leave the government [*public sector*]. Being employed in the government is not conducive for everyone to be there for a lifetime; turnover of staff is very high across all specialities, including eye care.’ (ID022, 45 years old, Female, Eyecare Deputy Director)

**Continuous professional development:** Optometrists expressed the need for more support in continuous professional development (CPD) to keep up to date with the current trends in the management of KC. Some optometrists have obtained qualifications such as ocular therapeutics and diagnostics certifications; however, the sector in which they practise needs to recognise this added skill in providing care to patients with KC. One of the respondents expressed the following:

‘We personally grab such studying opportunities from outside and at our own costs. There is no support.’ (IDO02, 38 years old, Female, Optometrist)

Preferences for continuous professional development activities tend to be given to other departments as expressed by the following participant:

‘… most of the time, the priorities are given to the nursing department.’ (IDO09, 53 years old, Female, Operations Manager)

Several participants highlighted the need for CPD, believing it might improve their KC management capacity. Furthermore, it has been noticed that training opportunities are more limited because of the coronavirus disease 2019 (COVID-19) pandemic:

‘… I would not say we are fully competent to fit contact lenses since we have not done it in about ten years. A refresher course would do.’ (IDO09, 53 years old, Female, Operations Manager)

‘We have not had any training opportunities since COVID-19.’ (IDO03, 55 years old, Male, Supply Chain Manager)

#### Theme 2: Infrastructural capacity towards keratoconus management

Infrastructural capacity was primarily centred around clinical equipment in the optometry department. Equipment and consumables for KC management were either unavailable, broken or poorly maintained.

**Equipment and space availability:** Some eye care facilities needed essential equipment and consumables for managing KC, such as rigid gas-permeable contact lens fitting sets and corneal topographers. Two of the participants expressed the following:

‘Yes, the minimum equipment for basic spectacle refraction is there. Also, regarding low-vision equipment, I think we have enough resources. We do not have enough equipment for paediatrics, and we have nothing for contact lenses.’ (IDO04, 32 years old, Male, Optometrist)

‘Mainly, we are lacking the equipment. We do not have the fitting set, RGPs, and contact lenses. As optometrists, we can manage patients with keratoconus theoretically, but we do not have the equipment.’ (IDO05, 27 years old, Male, Optometrits)

Besides the lack of ophthalmic equipment limiting KC management, several participants also mentioned the issue of space. One participant highlighted the following:

‘… The major limitations are the infrastructure and equipment and the space to do all this.’ (IDO08, 49 years old, Male, Supply Chain Manager)

Several participants expressed that they share the available space in the clinic with other units and departments, which hinders their operational capacity towards KC management. When asked about the integration of the eye clinic with other teams, one participant mentioned the following:

‘… I think it is not ideal, and we would like something that is more organised. Since we lack equipment and space, I do not think all this would be possible.’ (IDO09, 53 years old, Female, Operations Manager)

Several facilities are forced to refer patients with KC to other eye clinic facilities, including university teaching clinics in the province because they do not provide the services required to manage this condition because of a shortage of eye care personnel, a lack of specialist equipment for KC management and limited working space:

‘The hospital does not use hard contact lenses, and the ophthalmologist does not perform the crosslinking technique here. You diagnose the patient, book an appointment at [*hospital name withheld*], and write a referral letter for them. State all your findings you have observed on the patient.’ (IDO04, 32 years old, Male, Optometrist)

**Ophthalmic equipment procurement:** The participants involved in the eye clinic expressed a great deal of dissatisfaction with the tedious procurement process for the ophthalmic equipment and consumables that are used in KC management:

‘We always submit what we need, but it never comes. They always say there is no budget for it. We send a list to our operational manager, and it goes to procurement.’ (IDO05, 27 years old, Male, Optometrist)

The participants further expressed that not all cadres of eye care are aware of the procurement process of ophthalmic equipment. This limits the cadres’ involvement in developing services necessary for KC management:

‘No, I am not familiar with the procedure since our supervisors do the process. I know the process includes a meeting to discuss the budget and equipment needed for the following year.’ (IDO02, 38 years old, Female, Optometrist)

‘I am not clear with the protocol they follow when purchasing equipment, but from my knowledge, they meet to discuss and advertise; they contact people supplying that equipment. They use their SCM procedures to buy their equipment.’ (IDO03, 55 years old, Male, Supply Chain Manager)

However, one of the participants involved in the administrative procurement process (Director) expressed the following:

‘First, there must be a need. Annually, we have a 3-year plan, where we take all the needs and prioritise them according to the 3-year plan. The highest priority will be in the first year, and the least being in the third year. This is then sent to health technologies services at Wentworth Hospital, where they will look at our plan and allocate the budget for the required items. They then give us a mandate for where they will procure it on our behalf, or we can procure it at the institutional level.’ (IDO12, 48 years old, Male, Supply Chain Manager)

**Ophthalmic equipment maintenance:** The participants reported that there were no in-house technicians to maintain ophthalmic equipment; any faulty equipment must be reported to the manufacturer through a tedious administrative process. This lengthy process results in delays and thus affects the functionality of the eye clinic, resulting in either the patient being requested to return later or referred to neighbouring hospitals:

‘The problem is that the procurement guidelines are done by the Department of Health [*Provincial and National*]. What I would change is the speed, especially the paperwork. Speed up the time it takes for the equipment maintenance.’ (IDO08, 49 years old, Male, Supply Chain Mnager)

‘We report it to the head of the department, who then instructs the process through stores, and there is usually a department within the hospital that deals with the repairing of the equipment; if they cannot fix it, they take it back to the company.’ (IDO04, 32 years old, Male, Optometrist)

One participant further expressed the need for a specific optical technician as this may improve the administrative repair process. The consensus with the hospital management is that ophthalmic equipment is only serviced when it is faulty instead of a regular maintenance schedule. The participants expressed the following:

‘Yes, if they can get a maintenance person who knows more about optometry equipment because most of the workers there do not know a lot about our equipment. It is general maintenance people.’ (IDO05, 27 years old, Male, Optometrist)

‘Regarding general servicing, they should not wait until the equipment has a fault for it to be serviced.’ (IDO16, 44 years old, Female, Optometrist)

The participants also expressed discontentment with the lengthy wait when ophthalmic equipment is being serviced or repaired, further limiting their operationality:

‘… on average, it takes long, about 6–18 months now, to be fixed.’ (IDO09, 53 years old, Female, Operations Manager)

Another participant’s response when asked about the servicing of equipment in the eye clinic, proving that the KZN DoH uses no universal maintenance plan for the servicing of ophthalmic equipment:

‘I do not know; they had never been serviced since I arrived for the past five years.’ (IDO05, 27 years old, Male, Optometrist)

#### Theme 3: Operational capacity towards keratoconus management

**Protocols and coordination:**.It was clear that there were no definite guidelines or protocols for the day-to-day running of the clinic and for managing patients with KC. Instead, the diagnosis was primarily based on keratometry readings and heavily reliant on the practitioner’s knowledge and experience:

‘There are no specific guidelines, but you use your knowledge to check the cornea and K-readings.’ (IDO02, 38 years old, Female, Optometrist)

‘No, we do not have a standard protocol for diagnosing; we check the K-readings.’ (IDO05, 27 years old, Male, Optometrist)

The issue of professional scope of practice was also expressed by some of the optometrists involved in the study, highlighting that ophthalmic nurses performed specific procedures that fall under the scope of practice of the optometrist, and thus, they feel underutilised. Based on the comments of some optometrists, it became apparent that district coordinators and management generally did not know much about eye health, particularly KC. Therefore, support and guidance from management needed to be improved. They also felt they needed to be more involved in important issues such as budgeting, operations and overall induction within the hospital. One participant, who had a mid-level role in the eye clinic, expressed the following when asked about the financial resources of the clinic:

‘I cannot answer because I do not even know the budget amount.’ (IDO04, 32 years old, Male, Optometrist)

Several facilities expressed that they work under the supervision of the ophthalmology department and perform specialist duties over essential visual examinations. Emphasis is placed on other optometry specialties, such as low vision, diabetic retinopathy and cataract management, while KC management has a lesser priority:

‘We align our services with ophthalmology. On Mondays, we mostly see diabetic patients where the ophthalmology department does the posterior segment of the eye examination for diabetic retinopathy. If the patient requires refraction, they refer them to us. Tuesday, we do glaucoma patients. Wednesday and Thursday, we do first-time patients. Fridays is paediatrics day. We also do low vision on Mondays and Fridays since we can run two clinics.’ (IDO16, 44 years old, Female, Optometrist)

Another participant further expressed the following:

‘We work with the doctors, it is what they want, and so there is not much we can change.’ (IDO08, 49 years old, Male, Supply Chain Manager)

**Funding of eye care:** The participants also raised the issue of budgeting. The facilities experience budget cuts yearly, and the budget allocation for eye care services needed to be increased to cover the demand for eye care services, such as spectacle provision and especially KC management and funding to recruit more optometrists on an ad hoc basis:

‘We have been told already that we cannot order equipment more than R5000.’ (IDO16, 44 years old, Female, Optometrist)

‘… the provincial budget has been cut down, so hospitals would also have budget cut downs.’ (ID022, 45 years old, Female, Deputy Director eyecare)

It is evident that eye care services were not considered part of primary health care services. One participant who is involved in the director position expressed the following:

‘… The Department of Health never has enough budget for anything. It is not articulated only to eye care services, as in now we have budget target of more than 5 billion for everything, and it is not enough, but adequate to provide primary basic care. Unfortunately, for non-chronic diseases (NCDs) and chronic diseases, we do not have a grant for them, you only work under equitable share, and eye care falls under equitable share.’ (IDO22, 45 years old, Female, Deputy Director eyecare)

Some other facilities experienced difficulties with basic provisions of eye care services:

‘For example, this weekend, we were taking services to the people or rather the community, the hospital could only afford to give out 50 spectacles to patients while there we about thousands of patients needing spectacles in that area.’ (ID011, 42 years old, Female, Operations Manager)

In some other hospitals, even if a budget is allocated for optometry services, it tends to focus more on spectacle provision and low-vision aids. In contrast, consumables for complex corneal fitting need to be addressed. One of the participants expressed the following:

‘R325 000 is only for this hospital optometry services for last year, and for this year, we are still waiting for the budget allocation. This money is only for spectacles and low vision devices.’ (IDO16, 44 years old, Female, Manager Operation)

While in some hospitals, the budget is shared with other allied health departments, as expressed by another participant:

‘Since we share the budget with other departments, e.g., theatre, they would prioritise things needed in the theatre other than the eye department.’ (IDO08, 49 years old, Male, Supply Chain Manager)

Other participants mentioned the following regarding the sharing of the budget:

‘… The budget includes optometry and audiology.’ (IDO09, 53 years old, Female, Operations Manager)

‘The eye clinic budget is joined with Audiology. It is never enough for both departments. I wish we could have individual budgets like other departments as both our equipment and devices we provide are in demand and expensive.’ (IDO20, 27 years old, Female, Optometrist)

## Discussion

The study investigated factors enabling KC management within the public sector in KZN. The study revealed that the majority of the eye care personnel were mostly black females, while in the supply chain department, there was an equal distribution in gender and ethnicity. The higher number of females in the optometry field when compared to males could be related to the high number of them enrolled in optometry training universities in SA, as reported in a study by Oduntan et al.^15^ The high number of females was also linked to them being more likely to accept posts in public compared to their counterparts, as previously noted.^16^

As suggested by the WHO, for patient-to-eye care personnel ratios of 10 and 4.4 for optometrists and ophthalmologists, respectively, the human capacity still needs to be met. However, there is also an uneven distribution of optometrists in the facilities. The shortage of human resources for eye care that negatively affects service delivery has also been reported in other African countries such as Ghana and Nigeria.^17,18^ It also affects the number of patients being tested each day as the chair time required for KC diagnosis and management is longer than usual, leading to patients returning, if at all, for further testing. This led to a need for more people responsible for screening and early detection of KC. The lack of ophthalmologists was also reported, which led to only one site in the study having a corneal clinic and being able to conduct crosslinking and surgery for patients diagnosed with KC or any corneal disease. The lack of this service in hospitals close to communities leads to patients having to wait for a long time, impacting their quality of life because of keratoconus being a progressive disease.

The optometrists in this study reported that they believe themselves to be competent in the diagnosis and management of patients with KC. However, they admitted to requiring refresher courses on advanced skills in order to deal with the latest developments within the scope of contact lens fitting and to provide adequate aftercare for these patients. Similar results were also found in a study conducted in another province in SA.^11^ The DoH in KZN should provide opportunities to upskill the eye care personnel in order to provide the minimum standard of clinical care to patients diagnosed with KC. These can be achieved by collaborating with the training institution in the province.

This study also reported a lack or shortage of clinical equipment necessary for the diagnosis and management of KC. Only one public sector facility reported that they fit contact lenses and perform surgeries as a treatment option for patients with KC. However, the contact lenses used and given to patients on this site are sponsored by NGOs. This is of concern as most of the patients in the province of KZN access healthcare via the public sector, and management of some of the ocular conditions, such as pellucid marginal degeneration, KC and other corneal diseases, requires contact lenses and corneal surgery, which should be offered at many more facilities.

The participants also reported that the procurement process for clinical equipment and equipment servicing is tedious and not adhered to. It would, therefore, be useful for end users of the clinical equipment, which in this case are optometrists, to receive some training in certain minor aspects of maintenance, for example, calibration of equipment and changing of instrument bulbs. As adequate functioning equipment is essential to provide comprehensive eye care services, the challenges faced in relation to equipment will need to be addressed by the DoH in KZN. This will aid in reducing the waiting period and increase the number of patients who get assisted when visiting public health sector facilities. Shortage of clinical equipment, lack of infrastructure and trained personnel have been reported previously in other studies conducted in KZN.^17,19,20^

A review of protocols and guidelines used to detect and manage patients with KC revealed that there is currently no standard protocol defined for the day-to-day running of any of the facilities.^21^ This has led to facilities having different operational systems for eye care delivery, a finding not unique to KZN, as similar results have been reported in previous studies in other provinces.^10,22^ Therefore, there is a need to develop and implement a clear operational protocol that can be periodically evaluated to assess its effectiveness. Coordination of this recommendation may be a challenge in the absence of an eye care directorate, which was mentioned in this study. The option of creating a directorate for eye care should be considered at the provincial planning level.

Proper financing of health is crucial to making progress towards the delivery of eye care to everyone and is vital to meeting the universal health coverage as stipulated by the WHO. However, in this study, it was found that eye health is not integrated into the national or provincial health plans or budgets, an issue creating concerns at all facilities. Participants reported that the budget allocated for eye care is often shared with one of the allied departments and is never enough to cover essential services such as the provision of spectacles. One of the participants reported that the funds received from the national health departments are insufficient and limited, affecting the provision of eye care services, especially for conditions such as KC, which are increasing in prevalence. The shortage of funds for the provision of eye care limits the offering of comprehensive care for patients with KC as, in addition to being insufficient to procure human and material resources, it limits the implementation of health education, awareness and prevention services. Private–public partnerships should be considered as one of the options to address the financial challenges. These partnerships will aid in supporting training programmes for clinical staff and in the provision of equipment.

The leadership structure in the eye care department needs an urgent review as most of the participants reported that the current director of eye care or operations managers in most of the sites are often persons with insufficient understanding of specific areas of optometry. Most of the incumbents had a nursing background, and participants reported limitations in their understanding of requirements for diagnosis and management of conditions such as KC, which contributed to the challenges experienced by optometrists. Understanding that changes in leadership may not be feasible, the researchers suggest that the current cohort be trained in comprehensive care that includes requirements for ocular disease management, enabling them to advocate for eye care services by understanding the burden of VI in the province and districts. Private–public partnerships and sponsorships could be explored, even though they might not provide a complete solution to funding for KC patient care. They may provide substantial relief in the provision of eye care by sponsoring and donating much-needed equipment or contributing to some of the expenses that the public sector cannot afford.

### Limitations

Not all eye care districts and disciplines (ophthalmic nurses and ophthalmologists) who may have been able to provide further insight into the focus area were included in the study. Furthermore, there may have been a bias in the self-reported responses, which could have affected the reliability of the findings. These are noted as limitations of the study.

## Conclusion

This study highlighted the lack of a directorate for eye care and supports the need for the inclusion of eye care personnel in leadership roles within the province of KZN. Shortage of clinical equipment for KC diagnosis and management and uneven distribution of eye care personnel were observed. The budget currently allocated for the delivery of eye care services is inadequate; therefore, there is a need for a review of the budget and consideration of engaging in private–public partnerships. This will aid in the provision of comprehensive eye care to patients diagnosed with KC.
